# Metabolic Bile Acid Profile Impairments in Dogs Affected by Chronic Inflammatory Enteropathy

**DOI:** 10.3390/metabo13090980

**Published:** 2023-08-30

**Authors:** Rossana Comito, Emanuele Porru, Nicolò Interino, Matteo Conti, Rossella Terragni, Roberto Gotti, Marco Candela, Patrizia Simoni, Aldo Roda, Jessica Fiori

**Affiliations:** 1 Department of Medical and Surgical Sciences, Alma Mater Studiorum-University of Bologna, 40138 Bologna, Italy; rossana.comito2@unibo.it (R.C.); emanuele.porru2@unibo.it (E.P.); patrizia.simoni@unibo.it (P.S.); 2 Biostructures and Biosystems National Institute (INBB), 00136 Rome, Italy; aldo.roda@unibo.it; 3 Department of Chemistry “G. Ciamician”, Alma Mater Studiorum-University of Bologna, 40126 Bologna, Italy; nicolo.interino2@unibo.it; 4 Department of Public Health, Local Unit of Imola, Health Service of the Emilia-Romagna Region, 40026 Imola, Italy; matteo.conti@ausl.imola.bo.it; 5 Veterinary Clinic dell’Orologio/Veterinary Oncology Center, 40100 Bologna, Italy; terragni.rossella@gmail.com; 6 Department of Pharmacy and Biotechnology, Alma Mater Studiorum-University of Bologna, 40126 Bologna, Italy; roberto.gotti@unibo.it (R.G.); marco.candela@unibo.it (M.C.)

**Keywords:** bile acids, chronic inflammatory enteropathy dogs, faecal oxo-BAs, LC-MS/MS

## Abstract

Bile acids (BAs), endogenous acidic steroids synthetized from cholesterol in the liver, play a key role in the gut–liver axis physiopathology, including in hepatotoxicity, intestinal inflammatory processes, and cholesterol homeostasis. Faecal Oxo-BAs, relatively stable intermediates of oxidation/epimerization reactions of the BA hydroxyls, could be relevant to investigating the crosstalk in the liver–gut axis and the relationship between diseases and alterations in microbiota composition. A paucity of information currently exists on faecal BA profiles in dogs with and without chronic inflammatory enteropathy (CIE). Comprehensive assessment of 31 molecules among faecal BAs and related microbiota metabolites was conducted with high-performance liquid chromatography tandem mass spectrometry (HPLC-MS/MS). Odds ratios (ORs) for associations of BAs with CIE were estimated using logistic regression. Principal component analysis was performed to find differences between the control and pathological dogs. Higher levels of primary BAs and muricholic acids, and lower levels of secondary BAs were found in pathological dogs. Higher concentrations in faecal oxo-metabolites were associated with the absence of CIE (OR < 1). This study shows a marked difference in faecal BA profiles between dogs with and without CIE. Further research will be needed to better understand the role of oxo-BAs and muricholic acids in CIE dogs.

## 1. Introduction

Controlling the pool size and flux of bile acids (BAs) in the enterohepatic circulation is necessary for physiological functions and avoiding accumulation and toxic effects in vertebrates. BAs are secreted into the small intestine from the liver in a conjugated form with glycine or taurine. BAs are transformed via several metabolic pathways by intestinal microbiota. Firstly, BAs are deconjugated in the side chain to form free BAs that are substrates for different microbiota enzymes. Dehydroxylation, dehydrogenation and epimerization reactions lead to the formation of secondary (deoxycholic acid, lithocholic acid) and oxo metabolites.

Conjugated BA intestinal absorption mainly occurs in the terminal ileum by an active transport, while free BA absorption occurs along the entire intestinal tract by passive absorption. The efficiency of these processes is a critical step for recycling and BA homeostasis. BA synthesis in liver is regulated by a complex mechanism mediated by the Farnesoid X receptor (FXR) [1]. The interaction of BAs with FXR leads to the inhibition of their own synthesis; this occurs thanks to a chain reaction resulting in regulation of the first and limiting step in BA synthesis (hydroxylation of cholesterol in position 7α, catalysed by 7α cytochrome P450 CYP7A1). Moreover, the BA–FXR axis is also able to regulate BA homeostasis by downregulation of the ASBT (Apical Sodium Dependent Bile Acid Transporter) [2], which is the major transporter responsible for efficient uptake of BAs in the terminal ileum. Finally, FXR is able to promote FGF-19 (Fibroblast growth factor 19 in humans) secretion in the portal circulation. It functions as a hormone, regulating BA synthesis, with effects on glucose and lipid metabolism.

It is well known that BA composition varies widely among different animal species. Cholic acid, deoxycholic acid, and chenodeoxycholic acid in their tauro-conjugated form are the main BAs detected in the gallbladder of healthy dogs (around 73%, 20%, and 6%, respectively, of the total BAs pool) [3]. Other BAs and metabolites produced by gut microbiota like muricholic acids (α-muricholic acid and hyocholic acid) and oxidated BAs have been detected in faecal samples [4,5]. 

The composition and size of the BA pool must be considered because of the specific activity of each BA on several receptors like FXR and TGR5 (Takeda G protein-coupled receptor 5, and a G-protein-coupled bile acid receptor, Gpbar1) and their involvement in several physiological pathways. Moreover, alterations to the composition or activity of the gut microbiota due to antibiotics, exercise, diet or other dysbiotic states perturb BA metabolism [6]. The fine balance among BAs and microbiota can be altered during Inflammatory Bowel Disease (IBD). BA metabolism is markedly dysregulated in IBD, particularly when the microbiota is disrupted [6]. In this regard, diarrhea in patients with IBD may be partially dependent on BA malabsorption [7], which in turn, is commonly responsible for deficiencies in fat-soluble vitamins (A, D, E, and K). Diet, in fact, plays a key role in IBD disease. The most common micronutrient deficiencies observed are vitamin D, folic acid, vitamin B12, and iron [8].

Generally, the common mechanism involved in preclinical models (rat, dog, mouse, hamster and rabbit) of IBD/intestinal inflammation is decreased ASBT expression, which affects the BA profile in mammals due to a reduction in the BA concentration in the enterohepatic circulation [9,10,11].

Chronic inflammatory enteropathy (CIE) is the currently preferred term to describe what was previously referred to as IBD in veterinary medicine. There is much evidence regarding the relationship between intestinal microbiota and metabolites in health and disease states [12]. The latest studies at the molecular level (gene expression) have discovered deep alterations in the intestinal microbial communities of dogs with gastro intestinal diseases [13] from acute diarrhea, idiopathic and inflammatory bowel disease [14,15] to exocrine pancreatic insufficiency [16].

Together with microbiota alterations (disbyosis), recent studies have reported an imbalance in the BA composition, i.e., significant decreases in secondary BA concentrations in dogs with CIE. This has been observed with various analytical technologies including untargeted and targeted metabolomic approaches. Specifically, dogs with CIE have evidence of intestinal inflammation, BA dysmetabolism and persistent diarrhea [17,18]. Giaretta and colleagues reported decreased ileal ASBT protein expression in dogs with CIE. Interestingly, there was a significant negative correlation in this study between the cumulative histopathology score in the ileum and ileal ASBT expression. Moreover, dogs with CIE also had increased primary BA concentration (e.g., chenodeoxycholic acid) in faeces compared with the control group of animals, suggesting BA dysmetabolism [17]. Moreover, secondary faecal unconjugated BAs were decreased in dogs with a steroid-responsive form of CIE. Upon treatment of dogs with prednisone, there was a drug-induced increase in the faecal unconjugated BA content. It was concluded that corticosteroids therapeutically manage canine CIE by affecting BAs dysmetabolism [18]. The reduced intestinal transit time could account for the reduced formation of secondary BAs because of a reduced exposure of conjugated primary BA to intestinal bacteria deconjugation and 7 dehydroxylation.

Wang et al. reported that CIE dogs showed reduced secondary BAs (lithocholic and deoxycholic acid) in the faeces, accompanied by intestinal microbial dysbiosis [19]. Treatment with a hydrolysed protein diet decreased the abundance of pathogenic bacterial species (e.g., *Escherichia coli* and *Clostridium perfringens*), and concomitantly increased the levels of secondary BAs. Notably, there was a quick and prolonged clinical response to the diet-based therapy in most dogs with CIE. Interestingly, the investigators found that levels of a secondary BA-producing bacteria (*Clostridium hiranonis*) were increased after dietary therapy, which was linked to the clinical remission found in dogs with CIE. However, the class of oxo-BA (or keto BA) have not been evaluated in CIE. This class of BAs is largely present in mammalian faeces and also in dogs [20]. Indeed, they account for approximately 30% of the total BAs pool in dogs [5], influencing the balance of total BA content, reabsorption and essentially their physiological role. 

The present work focuses on the complete evaluation of the faecal BA pool in CIE dogs considering primary and 29 gut microbiota products as secondary and oxo-BAs (31 total compounds). The BA concentrations were determined by high-performance liquid chromatography combined with mass spectrometry (HPLC-MS/MS). We conducted a multivariate analysis (i.e., principal component analysis, PCA) to investigate possible differences between the control and pathological groups considering the clear link among microbiota, inflammatory enteropathy and BAs.

## 2. Materials and Methods

### 2.1. Chemicals

Analytical standards of cholic acid (CA), chenodeoxycholic acid (CDCA), deoxycholic acid (DCA), ursodeoxycholic acid (UDCA), lithocholic acid (LCA), muricholic acids (α-, β-, ω-MUCA, HCA) and isotopically-labeled internal standards were purchased from Sigma-Aldrich (Saint Louis, MO, USA). 

Standards of 3,7,12-trioxo-5β-cholan-24-oicacid, 7α,12α-dihydroxy-3-oxo-5β-cholan-24-oic acid, 3α,12α-dihydroxy-7-oxo-5β-cholan-24-oic acid, 3α,7α-dihydroxy-12-oxo-5β-cholan-24-oic acid, 7α-hydroxy-3-oxo-5β-cholan-24-oic acid, 3α-hydroxy-7-oxo-5β-cholan-24-oic acid, 3,7-dioxo-5β-cholan-24-oic acid, 12α-hydroxy-3-oxo-5β-cholan-24-oic acid, 3α-hydroxy-12-oxo-5β-cholan-24-oic acid, 3,12-dioxo-5β-cholan-24-oic acid, 3-oxo-5β-cholan-24-oic acid, 3α,6α-dihydroxy-7-oxo-5β-cholan-24-oic acid, 3α-hydroxy-6,7-dioxo-5β-cholan-24-oic acid, 3α-hydroxy-6-oxo-5β-cholan-24-oic acid, and 3,6-dioxo-5β-cholan-24-oic acid were purchased from Steraloids (Newport, CA, USA).

Standards of 7α,12β-dihydroxy-3-oxo-5β-cholan-24-oic acid, 12β-hydroxy-3-oxo-5β-cholan-24-oic acid, 7β-hydroxy-3-oxo-5β-cholan-24-oic acid, 7β,12α-dihydroxy-3-oxo-5β-cholan-24-oic acid, 6α-hydroxy-3-oxo-5β-cholan-24-oic acid, and 6α,7α-dihydroxy-3-oxo-5β-cholan-24-oic acid were synthesized following a procedure reported in our previously published paper [20].

Isopropanol, methanol (CH_3_OH), and acetonitrile (ACN), all of HPLC grade (Lichrosolv^®^), were purchased from Merck (Darmstadt, Germany). Acetic acid (98% pure), formic acid (98% pure), and ammonium hydroxide (98% pure) were purchased from Fluka (Buchs, Switzerland). Water of HPLC-MS grade was produced using the depurative system Milli-Q Synthesis A 10 (Millipore, Molsheim, France). Other solvents were all of analytical grade.

Stock solutions of each analyte and IS were prepared in isopropanol at a concentration of 1 mg/mL and stored at −20 °C. These stock solutions were further diluted in isopropanol to obtain working solutions containing all the analytes used for calibration curves and they were stored at 4 °C. 

### 2.2. Faecal Sample Preparation

Faecal sample preparation was performed as described in our previous study [20]. Briefly, aliquots of wet faecal sample homogenate (300 mg) were extracted with 900 μL of isopropanol. The mixture was homogenized using a Millipimer. The homogenates were left stirring for at least 2 h, underwent a sonication bath for 20 min, and were finally centrifuged twice at 13,000 rpm. The supernatant was then diluted 1:10 (*v*/*v*) with a mixture consisting of 40% isopropanol in 15 mM ammonium acetate at pH 8.00, transferred to an autosampler vial and injected into the RP-HPLC ESI-MS/MS system. The choice to use no dried samples was made because it would alter the physiological concentrations, which vary as function of water content. Eye-detectable dry matter in faeces from diet was excluded during the sampling of aliquots taken for analysis.

The results obtained from the analysis expressed as μg/mL of extract, were converted to μg/g of wet faeces by applying the following formula:

C = C_0_ × (V/m) (1)

where C represents the concentration expressed as μg/g;

C_0_ represents the concentration expressed as μg/mL;

V represents the volume of isopropanol (in mL) used for the extraction; 

m represents the weight of wet faeces (in grams) subjected to extraction.

### 2.3. HPLC-ESI-MS/MS Conditions

The HPLC-MS analytical method was developed and validated by the same authors in previous studies [20,21]. Briefly, liquid chromatography was performed using a 2690 Alliance system (Waters, Milford, MA, USA). Analytical separation was achieved using a XSelect CSH C18 (5 μm, 150 mm × 2.0 mm i.d, Waters) column kept at a constant temperature of 40 °C throughout the analyses. The mobile phase consisted of HPLC grade water with 15 mM ammonium acetate at pH 8.00 (A component) and methanol (B component). Final separation was achieved at a 0.15 mL/min flow rate under gradient elution conditions: 40% B for 2 min, 40–55% B from 2 to 5 min, 55% from 5 to 10 min, 55–65% B from 10 to 20 min, 65–80% B from 20 to 30 min, and 90% B from 30 to 40 min. Re-equilibration at 40% B between analyses was achieved in 10 min, for a total run time of 50 min. The injected sample volume was 10 μL. The autosampler temperature was kept at a temperature of 7 °C. The column effluent was introduced into the ESI source (negative ionization mode), connected to a triple quadruple mass spectrometer (Quattro-LC, Micromass/Waters, Milford, MA, USA) operating in the multiple reaction monitoring (MRM) acquisition mode. The data were managed and processed using MassLinx V4.0 software (Waters).

Nitrogen was used as the nebulizer gas at a 276 L/h flow rate and as the desolvation gas at 649 L/h. The ion source block and desolvation temperatures were set at 130 °C and 180 °C, respectively. The capillary and cone voltages were 2.7 kV and 50 V, respectively. MRM chromatograms are reported in Figure S1 of the Supplementary Material. Table 1 summarizes the retention times and the MS/MS transitions of each single compound.

### 2.4. Study Population 

Faecal samples were collected from 16 healthy dogs (7 males and 9 females), and 16 dogs (8 males and 8 females) with CIE (inflammatory bowel disease), 1–15 years old. The dogs were hospitalized with a non-specific antibiotic-free diet for at least 2 weeks. Dogs always had unlimited access to fresh water.

In our study, the severity of disease was estimated using the canine chronic enteropathy activity index (CCEAI), which is based on the presence and severity of 9 factors including attitude/activity, appetite, vomiting, consistency of faeces, frequency of defecation, weight loss, serum albumin concentrations, ascites and peripheral edema, and pruritus [22] (Table S1a,b). We had one sample in the soft group (score 0–3), two in mild (score 4–5), seven in moderate (score 6–8), 4 in severe (score 9–11) and two in the critical group (score > 12).

### 2.5. Statistical Analysis

Univariate and multivariate analyses were performed using GraphPad Prism 8·0·2 software. The normality distribution of the variables was tested using the D’agostino–Pearson omnibus normality test. The normally distributed variables were compared by *t*-test and one-way ANOVA; otherwise, the Mann–Whitney and Kruskal–Wallis tests were used; the significance level was 95%. Multiple variable analyses were performed by analyzing the constructed correlation matrix using Spearman’s coefficient for all BAs quantified in faecal samples. The correlation between two variables was studied as a function of *p*-value: 0.01 ≤ *p*-value < 0.05 (*) was considered statistically significant, 0.001 ≤ *p*-value < 0.01 (**) was considered highly significant, *p*-value < 0.001 (***) was considered extremely significant. Moreover, we decided to report the variables with at least moderate correlation (|rs| ≥0.5). A correlation coefficient ≥0.7 was chosen as the threshold for determining a strong correlation. Logistic regression analysis, performed by using Stata 421.17.0.112, was used to show the impact of each variable on the odds ratio (OR) of the observed event of interest: presence or absence of CIE. Chemometric analysis was performed with the R-based software CAT (Chemometric Agile Tool). We performed a principal component analysis (PCA) to visualize the clustering of the two groups of patients (control and pathological) to find possible outliers. Data analysis was performed using all major quantified BAs logarithmically transformed and the “centered” function of the software. Q and T2 tests were used as statistical methods to detect possible outliers using the Influence plot, considering all PCs that explained a variance ≥5%. The confidence interval was settled at 95%. The discriminant variables were chosen by considering the value of the loadings (|loading| ≥ 0.3).

## 3. Results

### 3.1. Pathological Subjects

Firstly, we constructed a correlation matrix considering the major BAs quantified and the canine chronic enteropathy activity index. We did not find any correlation (|rs|≥ 0.5) between the value of the score and BAs. Table S2 in Supplementary Material shows the faecal BA levels quantified in dogs stratified by CIE status.

### 3.2. Pathological vs. Control Subjects

Data analysis was continued considering pathological vs. control subjects. Pathological subjects had significantly higher faecal levels of CA (*p*-value 0.017). CDCA was also higher (*p*-value 0.057) in pathological subjects than in the controls. On the other hand, faecal DCA and LCA values were higher in controls (*p*-value 0.896 and 0.137, respectively). Regarding other microbiota metabolites, significantly higher levels of β-MUCA (*p*-value 0.021), and lower levels of 3,12-dioxo-DCA and 3-oxo-LCA (*p*-value 0.007 and 0.017, respectively) were determined. No significant differences were found for other BAs. In order to understand the effect of CIE on the BA pool, we calculated the amounts of distinct BA groups (primary, secondary and oxo-BA) as well as the sum of non-oxo-BAs and total BAs. We found a significantly higher concentrations of faecal primary BAs (*p*-value 0.032) in CIE dogs than in the controls. Table 2 summarizes the faecal BAs levels quantified.

**Figure 1:**
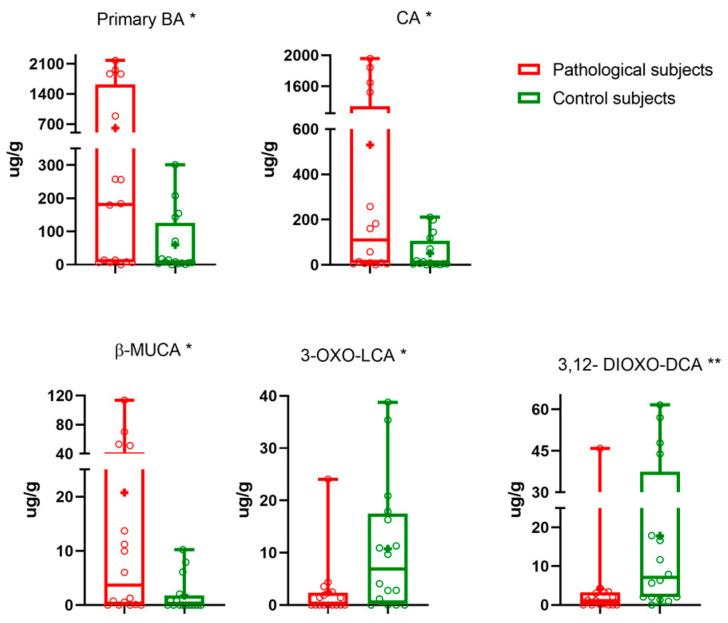
Statistically significantly different BAs. Median (line), mean (+) and the minimum and maximum. 0.01 ≤ *p*-value < 0.05 (*), 0.001 ≤ *p*-value < 0.01 (**).

The median (line), mean (+), interquartile range and the minimum and maximum values of faecal BA are reported in the box and whiskers plots for pathological (red), and control subjects (green) in Figure 1. Primary BAs are significantly different (*p*-value 0.032).

We performed several univariable logistic regressions using the presence/absence of CIE and the BAs (logarithmically transformed) between the two groups. Table 3 reports the cut-off of ROC (Receiver operating characteristic) curve chosen maximizing the sensitivity, OR, *p*-value and AUC (Area Under the Curve). Figure S2 in Supplementary Information shows the ROC curve for each BA investigated.

**Figure 2:**
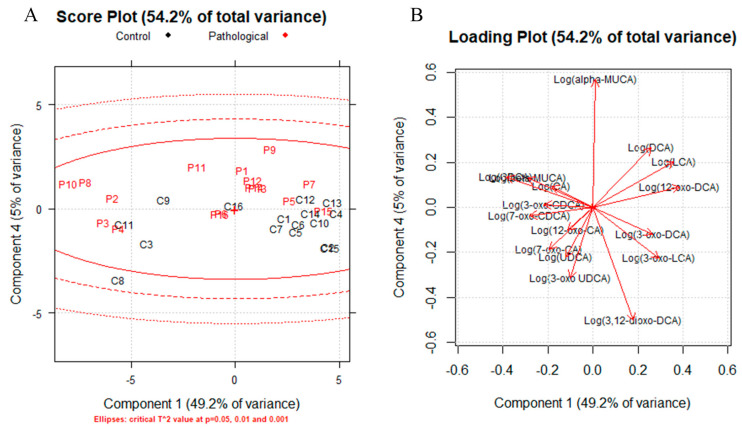
Score plot (**A**) for PC1 vs PC4, ellipses: critical T^2^ at p= 0.05, 0.01 and 0.001, controls are represented in black and pathological subjects in red Loading plot (**B**) for PC1 vs. PC4.

A correlation matrix was generated for all the samples, age, sex and dogs’ size, and we did not find any correlation (|rs|≥ 0.5) with BAs. Two correlation matrixes were generated to highlight the possible role of CDCA metabolites (Figure S3), one for controls and one for the pathological group. The results are reported in Table S3 in Supplementary Information. A positive correlation between β-MUCA and primary BAs was established for the pathological and control groups, while a negative correlation was found between β-MUCA and LCA. Principal component analysis (PCA) was performed to determine the presence of outliers and clusters using quantified BAs (logarithmically transformed). Firstly, we generated influence plots, one for each class (Figure S4 in Supplementary Information), using three and four components for the pathological group and controls, respectively, and any outlier was identified. Then, PCA was used to identify a multidimensional space cluster within the dataset. Figure 2 shows the score plot (a) and loading plot (b) (PC1 vs. PC4). Other PCs such as PC2 vs. PC4 were investigated (Figure S5).

The score plots showed a separation between the pathological subjects (red) and controls (black), with some patients overlapping the two classes. The loading plots allowed identification of the discriminating variables in the explored space (|loadings| ≥ 0.3), which are summarized in Table S4 in Supplementary Information. The main variables for the obtained clustering are α-MUCA, 3-oxo-UDCA and 3,12-dioxo-DCA for PC4; and LCA, 3-oxo-LCA, 12-oxo-DCA, CDCA and β-MUCA for PC1.

## 4. Discussion

The faecal composition of BAs and their metabolites is strongly correlated with the pathological state of the gastrointestinal tract due both to their malabsorption and their strict dependence on the metabolic activity and composition of the intestinal microbiota. The accurate and precise characterization of the BA pool can make an important contribution to the identification of faecal BA dysmetabolism and, more importantly, the determination of potential biomarkers of CIE. The LC-MS-MRM method, validated by the same authors, certainly represents an effective analytical tool for a completely reliable characterization and quantification of BAs and their related metabolites [20]. In fact, LC-MRM is able to determine a total of 31 molecules among BAs and their related microbiota metabolites. To the best of our knowledge, this is the first study to carry out chemometric studies with a BA pool of this size focusing of the quantification of BAs (μg/g of faeces) in dogs.

Only a few published studies investigate dog faecal BA profiles in health and disease; none of these dealt with quantifying oxo-BA metabolites and muricholic acids. To the best of our knowledge, the presence of muricholic acids in dogs has been reported in only one study [4]. Lin et al. [4] reported the formation of β-MUCA after incubation of UDCA in the liver microsomes of dogs; therefore, the 6β-hydroxylation can also occur in dogs.

The high levels of primary and conjugated BAs and low levels of secondary BAs in CIE faecal samples appeared to be consistent across the studies. The right proportions of primary and secondary BAs is an important regulator of gut homeostasis and is able to control inflammation processes. In fact, secondary BAs are reduced in dysbiosis associated with CIE [23]. Similarly, reduced amounts of secondary BAs and increased primary BAs are reported in faeces of humans with CIE and diarrhea-predominant irritable bowel syndrome [24]. Notably, the presence of diarrhea in CIE patients was suggested to be associated with an alteration in specific transport mechanisms within the gut, including those of BAs [25]. A decreased excretion of secondary BAs was detected in ulcerative colitis patients and attributed to a reduced transit time (diarrhea) and faecal pH as well as impaired microbial 7-α-dehydroxylase activity [26,27,28]. Considering the key role of 7-α-dehydroxylase in secondary BA formation, it could be a plausible explanation for the differences found in faecal secondary BA concentrations in CIE dogs.

Our results are in accordance with those previously published. An increase in primary faecal BAs and a decrease in secondary BAs were determined in CIE dogs. Furthermore, we found a clear increase in β-MUCA and a decrease in 3-oxo-LCA and 3,12-dioxo-DCA (*p*-value: 0.017 and 0.007, respectively). On the other hand, the presence of other metabolites, such as the 6-oxo derivatives from MUCA, was excluded as their concentrations were below the limit of detection of the method. Several studies have reported a change in gut microbial composition in the presence of CIE. For example, Honneffer et al. [29] reported a decrease in Fusobacteria and Bacteroidetes and an increase in Firmicutes among CIE dogs compared with healthy control dogs. *Faecalibacterium* spp., *Turicibacter* spp., *Escherichia coli*, *Streptococcus* spp., *Blautia* spp., *Fusobacterium* spp., and *Cl. hiranonis* are commonly altered in dogs with CIE [18]. *Cl. hiranonis* has been shown to play an important role in the conversion of primary BA to secondary BA and decreases in the faecal abundance of *Cl. hiranonis* have been reported in dogs with CIE [30]. These bacteria play a crucial role in BA metabolism and have a major influence on the gut metabolome. Dysbiosis can be considered a component of the pathophysiology of the chronic disease process since depletion of commensal groups and their respective immunoregulatory metabolites can impair the host’s ability to down-regulate the aberrant intestinal immune response [23]. Interestingly, some of these dysfunctional communications between the altered microbiota and intestinal immune system are mediated by metabolites, including the already mentioned secondary BAs and their derivates such as the 3 and 7-oxo-BA. 

Indeed, specifically, 3-oxo-LCA can bind the Retinoic-acid-receptor-related orphan nuclear receptor gamma (RORγt), which acts as a critical transcription factor for Th17 cell differentiation in chronic inflammation and autoimmune diseases [31] by acting as an inverse agonist [32,33]. Finally, recent studies [32,33] have shown that in a mouse model of colitis, the binding of RORγt decreases IL-17 production and Th17 cell number and attenuates intestinal inflammation. Among the oxo-BAs, we found significant differences in 3-oxo-LCA and 3,12-dioxo-DCA (*p*-value: 0.017 and 0.007, respectively), which were lower in CIE dogs than in controls. Thus, the significantly lower faecal concentration of 3- oxo-LCA could be consistent with the involvement of oxo-BA and this nuclear receptor family of transcription factors.

**Figure 3:**
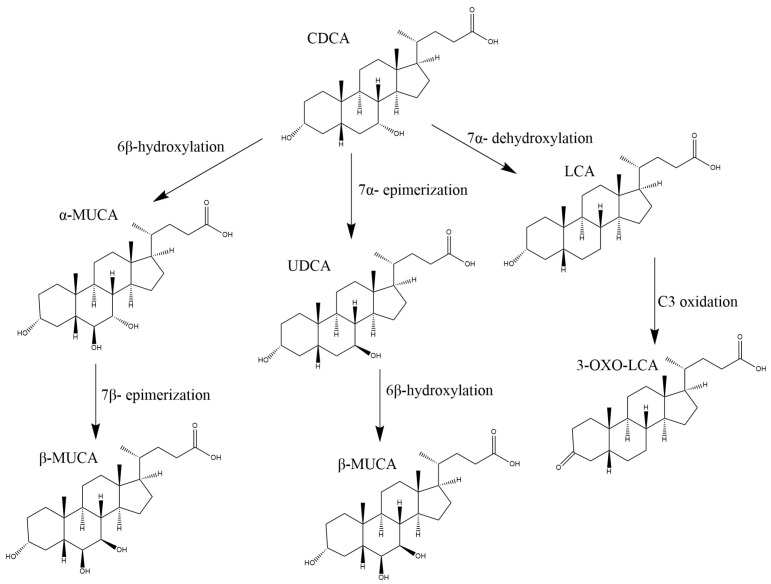
Physiological pathway for the production of CDCA metabolites.

Our data on the CDCA metabolites (Figure 3) β-MUCA and 3-oxo-LCA support the idea of an affected metabolism of primary BAs. The OR obtained from the logistic regression models showed that an increase in 3-oxo-LCA concentration is associated with the absence of CIE (85%, *p*-value 0.017). In accordance with this evidence, PCA showed the presence of two clusters, pathological and controls, with CDCA metabolites, the compounds responsible for the clustering (Figure 2).

To the best of our knowledge, this is one of the first studies in which faecal oxo-BAs and MUCAs are investigated in healthy and CIE dogs, representing attractive candidate biomarkers. These results are consistent with the previously published papers about dysbiosis, considering that even these molecules are metabolic products of commonly altered bacteria in dogs with CIE. One limitation of this study is the number of evaluated animals, even if the differences are statistically relevant and explained according with the already published literature. Further studies are necessary to explain the possible physiological role of 3-oxo-LCA and β-MUCA in CIE disease.

## Figures and Tables

**Table 1:** Retention times, lipid maps ID and the MS/MS transitions of each single compound included in the method.

Oxo-BA	Common Name	Retention Time (min)	Quantifier/Qualifier (*m*/*z*)	Lipid Maps ID
3,7,12-trioxo-5β-cholan-24-oic acid	trioxo-CA	8.6	[401.2]–[401.2]	LMST04010106
7α,12β-dihydroxy-3-oxo-5β-cholan-24-oic acid	12β-3-oxo-CA	12.5	[405.3]–[405.3]	Not reported
7β,12α-dihydroxy-3-oxo-5β-cholan-24-oic acid	3-oxo-UCA	12.9	[405.3]–[405.3]	Not reported
3α,6α-dihydroxy-7-oxo-5β-cholan-24-oic acid	7-oxo-HCA	17.86	[405.3]–[405.3]	LMST04010173
3α,12α-dihydroxy-7-oxo-5β-cholan-24-oic acid	7-oxo-CA	19.49	[405.3]–[405.3]	LMST04010184
3α,7α-dihydroxy-12-oxo-5β-cholan-24-oic acid	12oxo-CA	20.85	[405.3]–[405.3]	LMST04010176
3,7-dioxo-5β-cholan-24-oic acid	3,7-dioxo-CDCA	22.48	[387.3]–[387.3]	LMST04010136
6α,7α-dihydroxy-3-oxo-5β-cholan-24-oic acid	3-oxo-HCA	23.18	[405.3]–[405.3]	LMST04010145
3,12-dioxo-5β-cholan-24-oic acid	3,12-dioxo-DCA	23.18	[387.3]–[387.3]	LMST04010138
7β-hydroxy-3-oxo-5β-cholan-24-oic acid	3-oxo-UDCA	24.44	[389.3]–[389.3]	LMST04010162
3,6-dioxo-5β-cholan-24-oic acid	3,6-dioxo-HDCA	24.10	[387.3]–[387.3]	LMST04010134
7α,12α-dihydroxy-3-oxo-5β-cholan-24-oic acid	3-oxo-CA	25.8	[405.3]–[405.3]	LMST04010443
6α-hydroxy-3-oxo-5β-cholan-24-oic acid	3-oxo-HDCA	25.92	[389.3]–[389.3]	LMST04010158
3α-hydroxy-6-oxo-5β-cholan-24-oic acid	6-oxo-HDCA	26.32	[389.3]–[389.3]	LMST04010146
3α-hydroxy-7-oxo-5β-cholan-24-oic acid	7-oxo-CDCA	26.92	[389.3]–[389.3]	LMST04010150
12β-hydroxy-3-oxo-5β-cholan-24-oic acid	12β-3-oxo-DCA	27.77	[389.3]–[389.3]	LMST04010157
3α-hydroxy-12-oxo-5β-cholan-24-oic acid	12-oxo-DCA	28.67	[389.3]–[389.3]	LMST04010155
3α-hydroxy-6,7-dioxo-5β-cholan-24-oic acid	6,7-dioxo-CA	28.88	[403.2]–[403.2]	Not reported
7α-hydroxy-3-oxo-5β-cholan-24-oic acid	3oxo-CDCA	31.83	[389.3]–[389.3]	LMST04010161
12α-hydroxy-3-oxo-5β-cholan-24-oic acid	3oxo-DCA	32.58	[389.3]–[389.3]	LMST04010168
3-oxo-5β-cholan-24-oic acid	3oxo-LCA	38.22	[373.2]–[373.2]	LMST04010127
BA				
3α,6α,7β-trihydroxy-5β-cholan-24-oic acid	(ωMUCA)	20.27	[407.2]–[407.2]	LMST04010065
3α,6β,7α-trihydroxy-5β-cholan-24-oic acid	(αMUCA)	19.85	[407.2]–[407.2]	LMST04010066
3α,6β,7β-trihydroxy-5β-cholan-24-oic acid	(βMUCA)	20.87	[407.2]–[407-2]	LMST04010067
3α,6α,7α-trihydroxy-5β-cholan-24-oic acid	HCA	25.9	[407.2]–[407.2]	LMST04010064
3α,7β-dihydroxy-5β-cholan-24-oic acid	UDCA	26.28	[391.2]–[391.2]	LMST04010033
3α,6α-dihydroxy-5β-cholan-24-oic acid	HDCA	28.19	[391.2]–[391.2]	LMST04010024
3α,7α,12α-trihydroxy-5β-cholan-24-oic acid	CA	30.07	[407.2]–[407.2]	LMST04010001
3α,7α-dihydroxy-5β-cholan-24-oic acid	CDCA	35.15	[391.2]–[391.2]	LMST04010032
3α,12α-dihydroxy-5β-cholan-24-oic acid	DCA	35.91	[391.2]–[391.2]	LMST04010040
3α-hydroxy-5β-cholan-24-oic acid	LCA	39.56	[375.2]–[375.2]	LMST04010003

**Table 2:** All quantified BAs reported as mean ± standard error of mean, or median (interquartile range) as appropriate. *p* value: for the comparison between pathological vs. controls. Primary BAs: sum of CA and CDCA. Secondary BAs: sum of DCA, LCA, α-MUCA and β-MUCA. Oxo-BAs: sum of all oxo-BAs quantified. Non-oxo-BAs: sum of all primary and secondary BAs. Total BAs: sum of all quantified BA concentrations.

	Pathological Subjects (n = 16) μg/g	Control Subjects (n = 16) μg/g	*p*-Value
**Primary BAs**			
CA + CDCA	605 ± 211	8 [4–125]	0.032
CA	530 ± 189	51 ± 19	0.017
CDCA	3.6 [0.01–111.8]	0.01 [0.01–7.76]	0.057
**Secondary BAs**			
DCA + LCA + UDCA + α-MUCA + β-MUCA	594 [84–1106]	1001 [190–1537]	0.4230
DCA	496 [5–951]	699 [7–1216]	0.896
LCA	77.07 [0.09–203.2]	171 ± 36	0.137
UDCA	0.63 [0.01–5.55]	1.9 [0.01–4.49]	>0.999
α-MUCA	1.4 [0.8–3.5]	0.30 [0.01–4.00]	0.218
β-MUCA	3.65 [0.03–41.83]	0.01 [0.01–1.74]	0.021
**Oxo-BAs**	79 [35–411.4]	216 ± 40	0.386
12-oxo-CA	0.41 [0.01–4.06]	0.56 [0.01–2.57]	0.946
7-oxo-CA	12 [1–128]	27 ± 10	0.545
3-oxo-CDCA	0.43 [0.01–2.11]	0.01 [0.01–1.15]	0.126
7-oxo-CDCA	15 ± 5	0.01 [0.01–5.21]	0.198
12-oxo-DCA	16.02 [0.01–97.97]	94 ± 25	0.233
3-oxo-DCA	4.70 [0.01–40.25]	48 ± 13	0.117
3,12-dioxo-DCA	1.55 [0.01–3.24]	7 [2–37]	0.007
3-oxo-LCA	0.01 [0.01–2.36]	10± 3	0.017
3-oxo-UDCA	0.01 [0.010–0.36]	0.01 [0.01–0,01]	0.434
**Non-oxo-BAs**	1027 [682–2221]	879 [264–1426]	0.361
**Total BAs**	1102 [733–3041]	1076 [407–1723]	0.539

**Table 3:** OR, *p*-value, AUC, cut-off, sensitivity and specificity for each logistic regression model created. * BAs are logarithmically transformed.

BA *	OR (95% CI)	*p*-Value	Cut-Off	Sensitivity-Specificity	AUC (95% CI)
β-MUCA	3.67 (0.85–15.84)	0.082	−4.60	75–62.5%	0.73 (0.56–0.90)
3-oxo-LCA	0.15 (0.03–0.71)	0.017	1.03	81–69%	0.74 (0.56–0.91)
3,12-dioxo-DCA	0.04 (0.004–0.384)	0.005	1.73	93.75–62.50%	0.78 (0.62–0.95)
CA	3.86 (0.9–17)	0.078	2.16	75–56.25%	0.72 (0.54–0.90)
Primary BAs	2.83 (0.67- 12)	0.159	2.16	75–56.25%	0.72 (0.54–0.90)

## Data Availability

The data presented in this study are available on request from the corresponding author. The data are not publicly available due to privacy concerns.

## Supplementary Materials

The following supporting information can be downloaded at: https://www.mdpi.com/article/10.3390/metabo13090980/s1. Figure S1: Total ion current chromatogram reporting the separation of all 31 oxo-BAs investigated; Table S1a: Canine chronic enteropathy activity index (CCEAI); Table S1b: severity of factor in our IBD dogs; Table S2: Faecal BA levels quantified in dogs reported as median and interquartile range stratified by CIE status; Figure S2: ROC curves: (a) β-MUCA; (b) 3,12-dioxo-DCA; (c) 3-oxo-LCA; (d) CA; (e) primary-BA. All BA were logarithmically transformed; Figure S3: Paths to primary and secondary BAs and their oxo-derivatives; Table S3: Correlation matrix showing Spearman Coefficient and its *p*-value for the correlation between β-MUCA and all quantified BA. Only |rs|> 0.5 has been reported; Figure S4: Influence plot: Q vs. T2 Hotelling; (a) pathological and (b) controls; Figure S5: Score Plot and Loading Plot for PC2 vs. PC4; Table S4: Loadings for PC1, PC2, PC4. * BAs are logarithmically transformed.
