# Supplementary material for: Metabolic Bile Acid Profile Impairments in Dogs Affected by Chronic Inflammatory Enteropathy
Source: Metabolites. 2023 Aug 30;13(9):980. doi: 10.3390/metabo13090980 (PMC10535270; doi:10.3390/metabo13090980)
Supplement: Supplementary file 1 [file metabolites-13-00980-s001.zip › metabolites-2554260-supplementary.pdf]

Metabolic Bile Acid Profile Impairments in Dogs Affected by Chronic Inflammatory Enteropathy

Rossana Comito [1,2], Emanuele Porru [1,2], Nicolò Interino [3], Matteo Conti [4], Rossella Terragni [5], Roberto Gotti [6], Marco Candela [6], Patrizia Simoni [1], Aldo Roda [2,3] and Jessica Fiori [3,*]

[1] Department of Medical and Surgical Sciences, Alma Mater Studiorum-University of Bologna; 40138 Bologna, Italy; rossana.comito2@unibo.it (R.C.); emanuele.porru2@unibo.it (E.P.); patrizia.simoni@unibo.it (P.S.)
[2] Biostructures and Biosystems National Institute (INBB), 00136 Rome, Italy; aldo.roda@unibo.it
[3] Department of Chemistry "G. Ciamician", Alma Mater Studiorum-University of Bologna, 40126 Bologna, Italy; nicolo.interino2@unibo.it
[4] Department of Public Health, Local Unit of Imola, Health Service of the Emilia-Romagna Region, 40026 Imola, Italy; matteo.conti@ausl.imola.bo.it
[5] Veterinary Clinic dell'Orologio/Veterinary Oncology Center, 40100 Bologna, Italy; terragni.rossella@gmail.com
[6] Department of Pharmacy and Biotechnology, Alma Mater Studiorum-University of Bologna, 40126 Bologna, Italy; roberto.gotti@unibo.it (R.G.); marco.candela@unibo.it (M.C.)
* Correspondence: jessica.fiori@unibo.it; Tel.: +39-051-2099742; Fax: +39-051-2099734

Figure S1. Total ion current chromatogram reporting the separation of all 31 oxo-BAs investigated. (1) α-MUCA; (2) ω-MUCA; (3) β-MUCA; (4) HCA; (5) CA; (6) 12β,3-oxo-CA; (7) 3-oxo-UCA; (8) 7-oxo-HCA; (9) 7-oxo-CA; (10) 12-oxo-CA; (11) 3-oxo-HCA; (12) 3-oxo-CA; (13) UDCA; (14) HDCA; (15) CDCA; (16) DCA; (17) 3-oxo-UDCA; (18) 3-oxo-HDCA; (19) 6-oxo-HDCA; (20) 7-oxo-CDCA; (21) 12β, 3-oxo-DCA; (22) 12-oxo-DCA; (23) 3-oxo-CDCA; (24) 3-oxo-DCA; (25) 3,7-dioxo-CDCA; (26) 3,12-dioxo-DCA; (27) 3,6-dioxo-HDCA; (28) trioxo-CA; (29) 6,7-dioxo-CA; (30) 3-oxo-LCA; (31) LCA

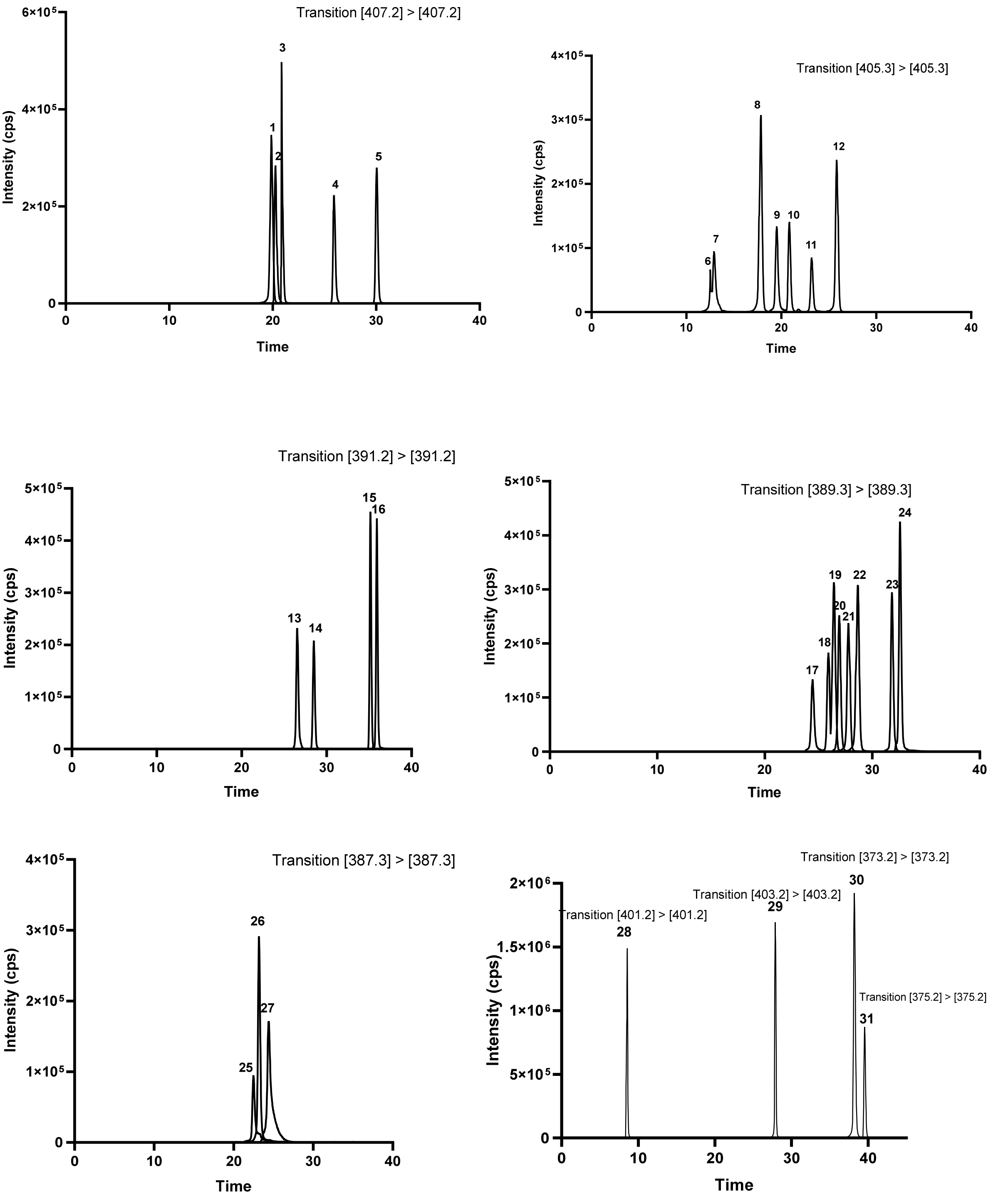

Table S1a. Canine chronic enteropathy activity index (CCEAI); S1b. severity of factor in our IBD dogs

a, Canine chronic enteropathy activity index (CCEAI)

Factor	0	1	2	3
a) Attitude/activity	normal	slightly decreased	moderately decreased	severely decreased
b) Appetite	normal	slightly decreased	moderately decreased	severely decreased
c) Vomiting	normal	mild (1x/week)	moderate (2–3x/wk)	severe (>3x/wk)
d) Consistency of feces	normal	slightly soft feces	very soft feces	watery diarrhea
e) Frequency of defecation	normal	slightly increased (2–3x/d) or fecal blood, mucus or both	moderately increased (4–5x/d)	3 severely increased (.53/d)
f) Weight loss	none	mild (<5%)	moderate (5–10%)	severe (>10%)
g) Serum albumin concentrations	albumin >20g/L	albumin 15–19.9 g/L	albumin 12–14.9 g/L	albumin <12 g/L
h) Ascites and peripheral edema	none	mild ascites or peripheral edema	moderate amount of ascites/ peripheral edema	severe ascites/pleural effusion and peripheral edema
i) Pruritus	no pruritus	occasional episodes of itching	regular episodes of itching, but stops when dog is asleep	dog regularly wakes up because of itching

b, severity of factor in our IBD dogs

ID	a	b	c	d	e	f	g	h	i	score
1	0	0	0	2	1	0	0	0	0	3
2	1	1	3	3	2	2	1	0	1	16
3	2	1	0	2	1	1	1	1	1	10
4	1	1	0	2	1	0	0	0	0	5
5	2	2	0	2	1	2	1	0	1	11
6	1	2	0	3	2	3	1	0	2	14
7	1	2	2	2	1	2	0	0	0	10
8	1	2	0	2	1	1	0	0	0	7
9	0	1	0	2	1	0	0	0	0	4
10	0	0	0	3	2	3	0	0	0	8
11	0	0	0	3	2	3	0	0	0	8
12	0	2	0	2	1	2	1	0	0	8
13	1	1	2	2	1	1	0	0	0	8
14	0	1	2	2	1	2	0	0	0	8
15	0	0	0	2	2	0	0	0	0	4
16	2	2	2	2	1	1	1	0	0	11

Table S2. Faecal BA levels quantified in dogs reported as median and interquartile range stratified by CIE status.

Pathological (n=16) µg/g	Normal (score 0-3) n=1	Mild (score 4-5) n=2	Moderate (score 6-8) n=7	Severe (score 9-11) n=4	Critical (score >12) n=2
**Primary BA**					
CA + CDCA	6.5	8 [7-9]	257 [184-1959]	10 [2-1401]	532 [179-885]
CA	5.3	7 [5-9]	257 [160-1842]	10 [2-1239]	430 [58-803]
CDCA	1.2	1 [0-2]	24 [0-334]	0 [0-162]	102 [82-122]
**Secondary BA**					
DCA+LCA+ UDCA+ α-MUCA+ β-MUCA	476.5	1019 [969-1069]	567 [78-3803]	364 [28-994]	1693 [19-3368]
DCA	417.7	828 [786-869]	513 [5-3466]	256 [8-854]	1595 [3-3187]
LCA	57.7	188 [96-281]	45 [0-320]	55.7 [0.5-131.0]	75 [0-149]
UDCA	0.1	0.5 [0-1.0]	0 [0-6]	1.50 [0.07-6.37]	17 [4-29]
α-MUCA	0.9	2 [1-2]	3 [1-5]	1.2 [0.2-17.9]	0.5 [0.3-0.7]
β-MUCA	0.1	0.3 [0.0-0.6]	14 [6-53]	0 [0-53]	6 [1-11]
**Oxo-BA**					
12-oxo-CA	0	0.4 [0.0-0.9]	3.8- [0.3-14.3]	0 [0-23]	0.4 [0.0-0.8]
7-oxo-CA	1.3	0.7 [0.0-1.5]	90 [11-223]	1 [0-258]	46 [13-80]
3-oxo-CDCA	0	0	1.6 [0.8-6.7]	0 [0-11]	1.1 [0.1-2.1]
7-oxo-CDCA	0	0	19 [2-31]	0 [0-32]	30 [5-56]
12-oxo-DCA	2	151 [24-278]	8 [0-109]	17 [0-58]	83.6 [0.1167.1]
3-oxo-DCA	3.4	126 [6-245]	7 [0-48]	0.7 [0.0-12.8	43 [0-87]
3,12-dioxo-DCA	0	68 [0-136]	1 [0-3]	1.6 [0.3-3.7]	1 [0-3]
3-oxo-LCA	0	30.00 [0-60]	0 [0-3.5]	0.7 [0.0-1.8]	0.7- [0.0-1.4]
3-oxo-UDCA	0.4	0	0	0.1 [0.0-2.9]	0.4 [0.2-0.7]
**Non-oxo-BA**	483.0	1027 [976-1078]	1948 [824-4061]	880 [160-1759]	2226 [904-3547]
**Total BA**	490.1	1403 [1006-1799]	2110 [860-4349]	944 [173-2107]	2434 [989-3879]

Figure S2. ROC curves: (a) β-MUCA; (b) 3,12-dioxo-DCA; (c) 3-oxo-LCA; (d) CA; (e) primary-BA. All BA were logarithmically transformed.

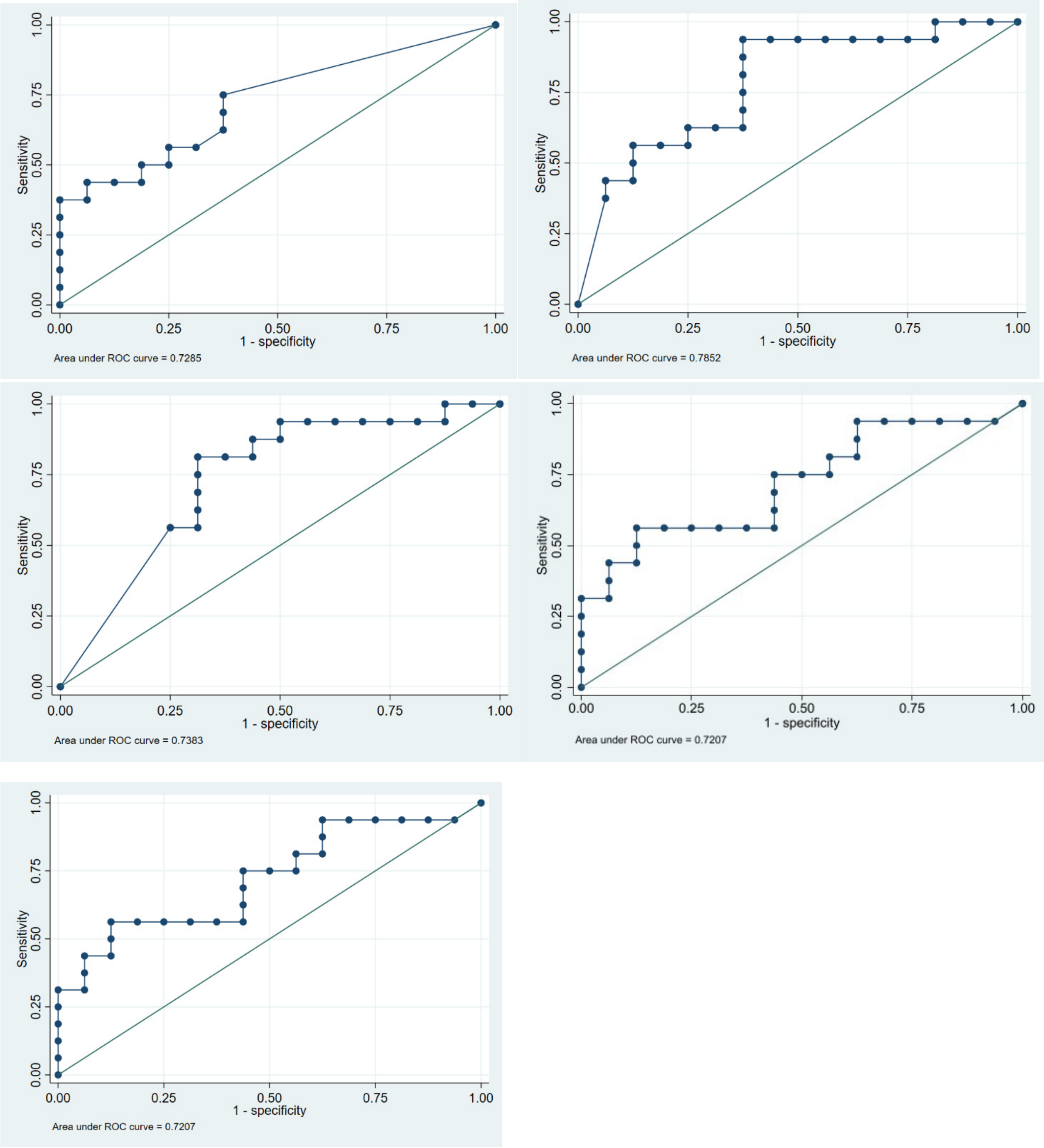

Figure S3. Paths to primary and secondary BAs and their oxo-derivatives

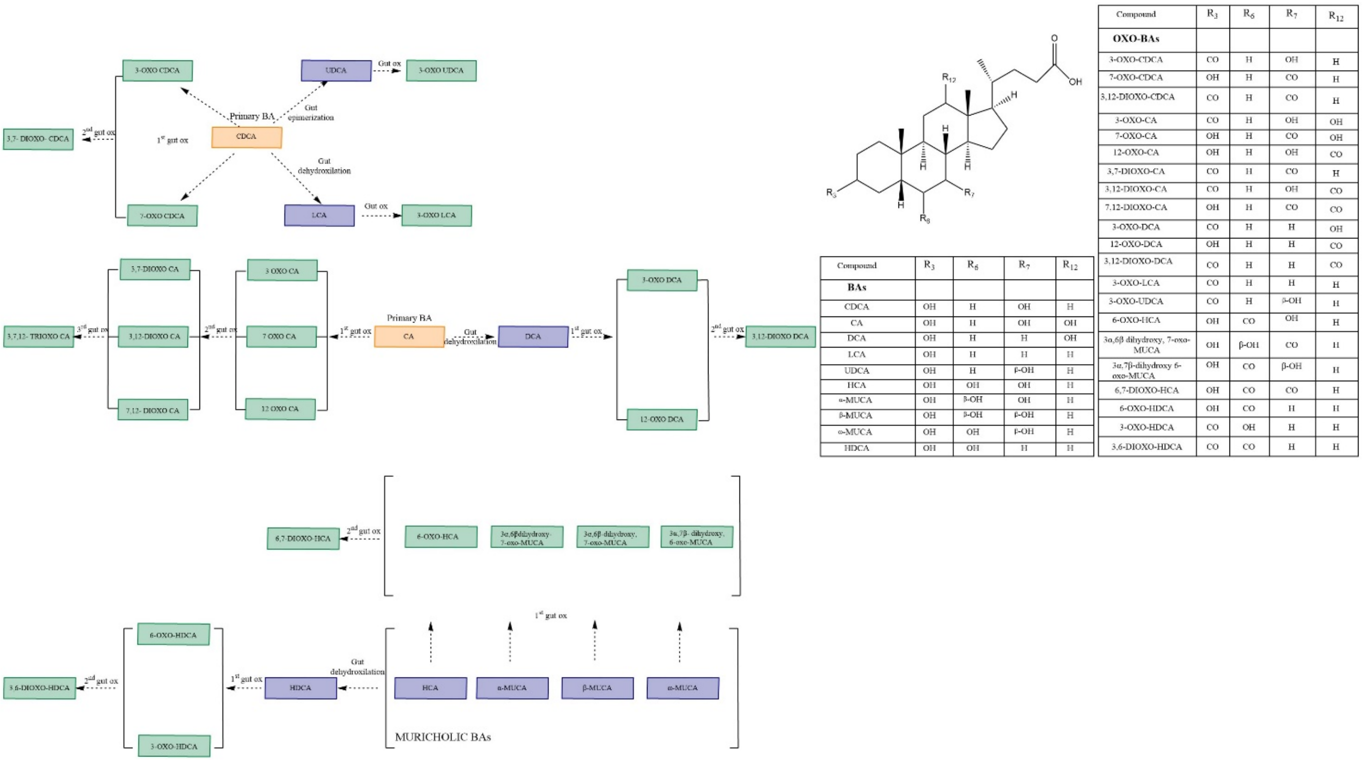

Compound	R_3	R_6	R_7	R_{12}
**BAs**				
CDCA	OH	H	OH	H
CA	OH	H	OH	OH
DCA	OH	H	H	OH
LCA	OH	H	H	H
UDCA	OH	H	β-OH	H
HCA	OH	OH	OH	H
α-MUCA	OH	β-OH	OH	H
β-MUCA	OH	β-OH	β-OH	H
ω-MUCA	OH	OH	β-OH	H
HDCA	OH	OH	H	H

Compound	R_3	R_6	R_7	R_{12}
**OXO-BAs**				
3-OXO-CDCA	CO	H	OH	H
7-OXO-CDCA	OH	H	CO	H
3,12-DIOXO-CDCA	CO	H	CO	H
3-OXO-CA	CO	H	OH	OH
7-OXO-CA	OH	H	CO	OH
12-OXO-CA	OH	H	OH	CO
3,7-DIOXO-CA	CO	H	CO	H
3,12-DIOXO-CA	CO	H	OH	CO
7,12-DIOXO-CA	OH	H	CO	CO
3-OXO-DCA	CO	H	H	OH
12-OXO-DCA	OH	H	H	CO
3,12-DIOXO-DCA	CO	H	H	CO
3-OXO-LCA	CO	H	H	H
3-OXO-UDCA	CO	H	β-OH	H
6-OXO-HCA	OH	CO	OH	H
3α,6β dihydroxy, 7-oxo-MUCA	OH	β-OH	CO	H
3α,7β-dihydroxy 6-oxo-MUCA	OH	CO	β-OH	H
6,7-DIOXO-HCA	OH	CO	CO	H
6-OXO-HDCA	OH	CO	H	H
3-OXO-HDCA	CO	OH	H	H
3,6-DIOXO-HDCA	CO	CO	H	H

Table S3. Correlation matrix showing Spearman Coefficient and its p-value for the correlation between β-MUCA and all quantified BA. Only rs> 0.5 has been reported.

Pathological subjects			Control subjects		
BA	Spearman Coefficient	p-value	BA	Spearman Coefficient	p-value
CDCA	0.626	0.009	LCA	-0.625	0.011
3-OXO-CDCA	0.887	<0.0001	3-OXO-LCA	-0.697	0.004
7-OXO-CDCA	0.856	<0.0001	DCA	-0.556	0.027
CA	0.927	<0.0001	12-OXO-DCA	-0.663	0.005
12-OXO-CA	0.680	0.004	3-OXO-DCA	-0.688	0.003
7-OXO-CA	0.958	<0.0001	3,12-DIOXO-DCA	-0.541	0.031
			CDCA	0.781	<0.001
			3-OXO-CDCA	0.729	0.001
			7-OXO-CDCA	0.715	0.002
			CA	0.686	0.003
			7-OXO-CA	0.701	0.002

Figure S4. Influence plot: Q vs T2 Hotelling; a) pathological and b) controls

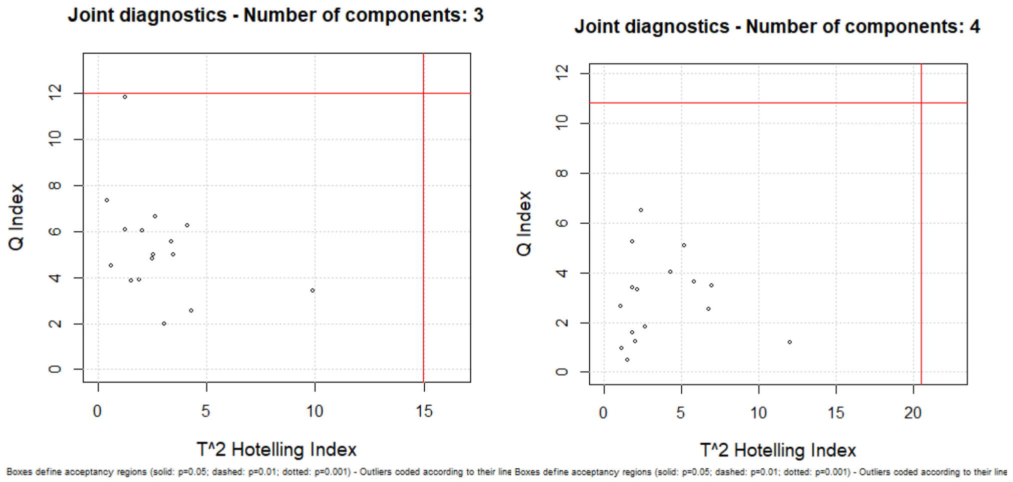

Figure S5. Score Plot and Loading Plot for PC2 vs PC4

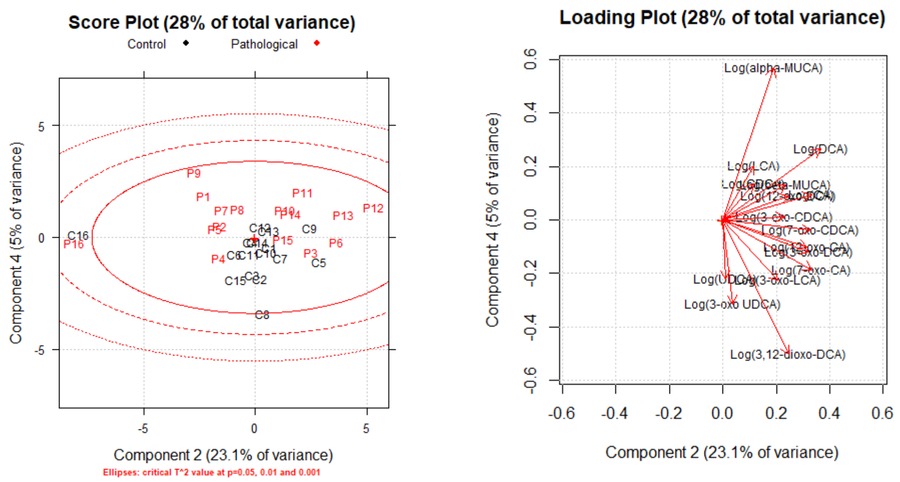

Table S4. Loadings for PC1, PC2, PC4. * BAs are logarithmically transformed

Variable*	PC1	PC2	PC4
LCA	0.36		
3-oxo-LCA	0.30		
DCA		0.37	
12-oxo-DCA	0.39		
3-oxo-DCA		0.32	
3,12-dioxo-DCA			-0.50
CDCA	-0.38		
7-oxo-CDCA		0.33	
3-oxo-UDCA			-0.31
CA		0.33	
7-oxo-CA		0.33	
12-oxo-CA		0.31	
α-MUCA			0.57
β- MUCA	-0.3		
